# ‘Emptiness filled with love’: A reflexive thematic analysis of chemsex trajectories among gay, bisexual and other men who have sex with men in Almaty, Kazakhstan, using a life course framework

**DOI:** 10.1111/add.70454

**Published:** 2026-04-24

**Authors:** Nikolay Lunchenkov, Nadezhda Cherchenko, Elena German, Susanna Rinne‐Wolf

**Affiliations:** ^1^ TUM School of Social Sciences and Technology Technical University of Munich Munich Germany; ^2^ Health and Capacity Building Department, Eurasian Coalition on Health, Rights, Gender and Sexual Diversity Tallinn Estonia; ^3^ Department of Scientific Management and Education Republic Scientific and Practical Centre of Mental Health Almaty Kazakhstan; ^4^ Chair for Public Health and Health Services Research Institute for Medical Information Processing, Biometry and Epidemiology, Pettenkofer School of Public Health, Ludwig‐Maximilians‐Universität Munich Germany

**Keywords:** chemsex, gay and bisexual men, Kazakhstan, life course theory, qualitative research, substance use trajectories

## Abstract

**Background and aims:**

The intentional use of psychoactive substances to enhance sexual experiences, known as chemsex, is associated with human immunodeficiency virus (HIV) transmission, psychological distress and social isolation among gay, bisexual and other men who have sex with men (GBMSM). While research has predominantly focused on high‐income countries, gaps remain in our understanding of the dynamics in Eastern Europe and Central Asia, where structural homophobia, restrictive drug policies and limited healthcare access uniquely shape chemsex‐related risks. There is limited research on how chemsex engagement evolves over time. This study aimed to identify the stages of chemsex engagement and examine how social, psychological and structural factors shape transitions between these stages among GBMSM in Kazakhstan.

**Methods:**

This study is a secondary analysis of data from a cross‐sectional qualitative study conducted in Almaty, Kazakhstan, between July and September 2023. Twenty‐one GBMSM who had engaged in chemsex within the past 12 months participated in semi‐structured interviews lasting between 60 and 90 minutes. All participants reported using mephedrone and/or alpha‐PHP (α‐Pyrrolidinohexiophenone). Deductive reflexive thematic analysis, guided by Life Course Theory, was used to reconstruct chemsex trajectories from retrospective participant accounts.

**Results:**

Five distinct themes related to different trajectory stages were developed. Initiation occurred through trusted social networks in intimate settings, taking the form of unplanned encounters that fulfilled emotional needs for connection and belonging. Maintenance was characterised by self‐imposed temporal and dosage limits; however, participants reported a decline in satisfaction with sober sex. Escalation involved a breakdown of protective boundaries, an increased frequency and quantity of use and a deeper involvement in chemsex social networks, despite mounting physical and psychological consequences. Dependence was characterised by a narrowed agency under escalating constraints, with substance use shifting from enhancing pleasure to coping with withdrawal, resulting in substantial impairment across life domains. Disengagement attempts described recursive trajectories with diverse recovery goals ranging from cessation to managed use. Sustained abstinence was rare and dependent on affirming care and supportive resources. Throughout all stages, structural stigma constrained individual agency, while chemsex networks provided crucial emotional safety that was unavailable in mainstream environments.

**Conclusions:**

Chemsex trajectories among gay, bisexual and other men who have sex with men in Kazakhstan represent dynamic processes shaped by the interaction between individual agency and structural constraints, in which substance use functions as an emotional regulation strategy and a social survival strategy in contexts of high stigma.

## INTRODUCTION

Chemsex—the intentional use of emerging psychoactive substances like mephedrone, gamma‐hydroxybutyrate (GHB)/gamma‐butyrolactone (GBL), crystal methamphetamine or α‐pyrrolidinohexanophenone (α‐PHP) to enhance sexual experiences is increasingly recognised as a global health concern among gay, bisexual and other men who have sex with men (GBMSM), which can be associated with HIV transmission [1, 2, 3]. Research has predominantly focused on high‐income countries, leaving significant gaps regarding its dynamics in Eastern Europe and Central Asia (EECA), including Kazakhstan, where structural homophobia, restrictive drug policies and limited healthcare access uniquely shape chemsex‐related risks [3, 4, 5, 6, 7, 8, 9, 10]. In addition to these epidemiological risks, chemsex is increasingly associated with psychological distress, social isolation and community‐reported deaths [4, 11, 12, 13, 14].

However, scholars have cautioned that exclusively framing chemsex through a risk lens may reinforce stigma and create barriers to help‐seeking [15, 16, 17, 18], whereas a substantial body of research emphasises its association with pleasure, intimacy, community building and resistance to heteronormative sexual scripts and homophobic social environments [19, 20, 21, 22, 23]. Rather than assuming chemsex is inherently problematic, we examine how trajectories unfold over time and apply theoretical frameworks to understand transitions in engagement patterns.

Despite recognising this complexity, current research primarily examines prevalence, health risks and harm reduction approaches [6, 24, 25, 26], but lacks insight into temporal dynamics. Although longitudinal studies document variation in trajectories [24, 25], few apply theoretical frameworks to explain these transitions. Understanding these temporal dynamics is essential for identifying intervention points, targeting support to specific stages and recognising that progression to dependence is not inevitable.

Life course theory (LCT) provides a framework for understanding substance use trajectories by emphasising the interplay between individual lives and broader historical, social and cultural contexts [26, 27]. LCT is organised around five key principles, (1) lifespan development; (2) agency; (3) time and place; (4) timing; and (5) linked lives, that examine how age, life events, relationships and turning points shape behavioural patterns [26, 27]. For GBMSM practicing chemsex in Kazakhstan, LCT offers particular analytical value because structural constraints fundamentally reshape agency, timing and linked lives. However, LCT was developed largely in Western, heteronormative contexts and may not fully capture queer life‐worlds under extreme structural constraints. Its emphasis on developmental sequencing can inadvertently privilege linear trajectories [26]. We, therefore, apply LCT flexibly, examining how structural forces in Kazakhstan fundamentally reshape agency, timing and linked lives, and focusing on how structural forces reshape core principles across all stages of chemsex engagement.

Building on our earlier qualitative work [28], this study uses LCT to understand how social, psychological and structural factors shape chemsex trajectories among GBMSM in Almaty, a conservative setting where punitive policies and pervasive homophobia create unique constraints on substance use patterns and recovery pathways.

## METHODS

Reporting methods and results the authors adhered to the Consolidated Criteria for Reporting Qualitative Research (COREQ) [29], which can be found in Data S1.

### Design

This study draws on data originally collected as part of qualitative research exploring chemsex practices among GBMSM in Almaty, Kazakhstan, using an adapted life course framework and deductive reflexive thematic analysis [27, 30, 31]. The original study explored the experiences, motivations and perceived benefits and risks of chemsex among GBMSM in Almaty, and to identify their demand for specific health and psychotherapeutic services. The original research questions focused on three areas: (1) understanding the psychological, social and interpersonal factors associated with chemsex engagement; (2) examining participants' risk perceptions and harm awareness; and (3) identifying what clinical and mental health services participants perceived as most needed (see Data S2). The current article represents a secondary, theory‐driven analysis of this dataset, applying LCT to examine temporal trajectories of chemsex engagement. A detailed description of the methods has been published elsewhere [28]. However, to provide sufficient context for the current analysis, key methodological aspects are summarised here.

### Study settings and participants

The study took place in Almaty, Kazakhstan's largest city and a key hub for lesbian, gay, bisexual, trans, queer+ (LGBTQ+) communities [7, 9]. Although relatively open by regional (Central Asian) standards, it remains shaped by conservatism, punitive drug laws and limited non‐stigmatising health services. Although same‐sex relations are not explicitly criminalised, the adoption of legislation restricting so‐called ‘LGBT propaganda’ in December 2025 has partially criminalised LGBTQ+ life. LGBTQ+ individuals face widespread discrimination and violence without legal protection, and anti‐LGBTQ+ rhetoric from political and religious leaders forces most to live underground [8, 32, 33]. Access to healthcare remains severely constrained by stigma and a lack of culturally competent providers, particularly in mental health [8, 9, 10].

Drug policies are highly repressive, for example, possession of mephedrone can result in imprisonment and compulsory registration, leading to lifelong exclusion [34, 35]. Treatment is coercive and abstinence‐based, with harm reduction focused on opioid users and neglecting GBMSM. Currently, only one community‐based organisation offers chemsex‐specific support. They provide safer use kits and LGBTQ+‐affirming mental health counselling. However, operating with minimal resources means their reach remains limited. The intersection of homophobia and drug criminalisation leaves GBMSM who engage in chemsex doubly marginalised, with almost no access to comprehensive, non‐stigmatising care. It is within this context of structural exclusion and growing political hostility that participants' experiences of chemsex must be understood.

### Data collection

Participants were recruited via community‐based organisations and digital platforms (Telegram, Hornet). These organisations provide HIV and sexual transmitted infections (STI) testing, harm reduction services and psychosocial support specifically for GBMSM. Eligibility criteria included: be over 18 years of age; identify as GBMSM; reside in Almaty; have engaged in chemsex within the past year; and be able to communicate effectively in Russian. Of the 39 individuals who initially expressed interest, 21 (54%) ultimately enrolled and participated in interviews.

The first author conducted 21 semi‐structured interviews (60–90 minutes) in Russian in a private room at a partner non‐government organisation (NGO) between July and September 2023. All interviews were audio‐recorded, transcribed verbatim and pseudonymised. Participants provided informed consent and received compensation (~USD 25). Ethical approval was obtained from Technical University of Munich (537/21 S‐NP) and Kazakh National Medical University (no. 3/139).

### Data analysis and theoretical framework

This study applies theory‐driven, reflexive thematic analysis through the lens of an adapted life course framework [27, 31]. We adopted a deductive approach to explore how participants reflected on their chemsex engagement over time. We use the term chemsex trajectory to refer to the evolving pattern of substance use and sexual behaviour across stages of initiation, maintenance, escalation, dependence and disengagement, as experienced and described by participants. These stages represent analytical constructs applied by the research team to organise temporal narratives, not self‐identified categories. Individual narratives often spanned multiple stages, and some participants were cycling between patterns. Although cross‐sectional in design, the study reconstructs trajectories through detailed retrospective accounts, consistent with established life course methodology [26, 27, 36].

This analysis represents a secondary examination of the dataset. An initial publication focused on motivations and risk perceptions [28]. During that analysis, we observed that participants spontaneously structured their narratives temporally, warranting deeper exploration given the absence of trajectory‐focused research in restrictive settings. Following initial peer review, we developed a fifth theme, disengagement, to capture participants' varied attempts to reduce or cease use, allowing us to examine recursive trajectories and diverse recovery goals more comprehensively.

To operationalise LCT, we coded for changing motivations across life phases (lifespan development), constrained choices and self‐imposed rules (agency), the influence of Kazakhstan's structural context (time and place), critical life events catalysing transitions (timing) and peer networks shaping trajectories (linked lives). Throughout the findings, we identify where each principle manifests in participants' accounts.

The five trajectory stages, derived from substance use literature [27, 37] served as sensitising concepts rather than fixed categories. We remained open to cyclical and non‐linear experiences. As all participants had engaged in chemsex within 12 months of interview, the analysis encompasses all phases including disengagement attempts. Although sustained disengagement exceeding 12 months was not represented, several participants described active recovery attempts, revealing recursive rather than linear trajectories.

Following Braun and Clarke [30, 31, 38], the analytic process was interpretive and iterative, proceeding through six steps. Although the team were familiar with the dataset from previous work, the first step involved re‐reading the transcripts and listening to some of the audio files to reacquaint ourselves with context, emotional tone and temporal shifts. In step 2, coding proceeded iteratively in *ATLAS.ti* [39]. We began by applying the five trajectory stages as broad organising categories to segment participants' narratives temporally. Within each trajectory stage, we, then, developed inductive codes to capture the specific content and processes participants described. These inductive codes included both descriptive codes capturing content (e.g. ‘first sex on drugs’, ‘self‐imposed limits’, ‘weekend‐only use’) and interpretive codes capturing process (e.g. ‘loss of control’, ‘chemsex‐exclusive socializing’, ‘normalisation of risk’). This enabled us to integrate theoretically guided attention to life‐course dynamics with openness to the complexity and diversity of lived experience. In step 3, we grouped related inductive codes into subthemes corresponding to different dimensions within each trajectory stage. For example, within the ‘initiation’ stage, codes related to social setting, peer trust and emotional needs were grouped into subthemes. In step 4, subthemes were checked for consistency and clarity, with some codes revised or merged to better reflect transitions between stages. In step 5, the final thematic map was agreed on by the research team and aligned with the adapted framework (see Data S3). The relationship between deductive trajectory stages, inductive subthemes and LCT principles is presented in Data S4. In step 6, representative data excerpts were integrated into the findings to illustrate each theme.

Throughout, we adhered to principles of reflexivity and theoretical coherence, acknowledging that our interpretation was shaped by our positionalities and LCT's theoretical commitments. This balance of deductive structure and inductive flexibility aligns with reflexive thematic analysis [30, 38]. Reflexivity was maintained through ongoing dialogue with co‐authors and chemsex researchers, particularly when participants account became emotionally challenging.

### Researcher personal statement

The lead author's positionality as a gay, Russian‐born medical doctor and qualitative researcher with personal and professional experience of sexual health and substance use in EECA shaped this study. His shared identity facilitated emotional safety and trust during interviews, enabling deeper data collection, while requiring careful reflexivity regarding potential biases. By the time this article was written, the lead author was enrolled in a doctoral program at the Technical University of Munich. The other authors, identifying as female and, therefore, holding outsider perspectives on GBMSM experiences, acknowledge the limitations inherent to their viewpoints.

This study is grounded in a critical realist ontology and constructivist epistemology. Although participants' experiences have tangible psychological and social consequences, they are also shaped by contextual, relational and discursive processes. Knowledge is co‐constructed through dialogue and interpretation, and meanings of sexualised substance use are understood as socially produced and embedded within systems of stigma, desire and regulation.

All authors subscribe to a harm reduction approach to substance use. We understand sexualised substance use as one avenue through which GBMSM navigate intimacy, sexual expression and emotional connection within contexts of structural stigma. We prioritise participant interpretation and resist pathologising frameworks that position chemsex as inherently deviant or disordered.

Although feedback from people with lived experience informed study design and recruitment, practical limitations prevented their full participation throughout the research process. The lead author's understanding evolved significantly during the study. Initially, his insider familiarity led him to emphasise pleasure and community. However, participants' accounts of escalating use, combined with the loss of a close friend, heightened his awareness of harmful trajectories while reinforcing the imperative to avoid pathologising all chemsex engagement. The female co‐authors and external consultants ensured that this shift did not result in overcorrection, insisting that attention be paid to pleasure, agency and constrained choice alongside harm. This collaborative reflexive process maintained analytical balance throughout.

## RESULTS

### Participants’ characteristics

The sample included 21 GBMSM in Almaty who had engaged in chemsex in the past 12 months. Most identified as gay (*n* = 14) and Kazakh (*n* = 13), with a median age of 26 (range: 20–36). All reported using mephedrone and/or α‐PHP, with varying group and episodic use patterns (see Table 1).

**TABLE 1:** Socio‐demographic characteristics of GBMSM participants (*n* = 21).

Characteristics	Value
Age (years)	
Range	20–36
Median	26
Sexual identity	
Gay	14 (67%)
Bisexual	5 (24%)
Other identities^a^	2 (9%)
Ethnicity	
Kazakh	13 (62%)
Russian or Ukrainian	6 (29%)
Kyrgyz or Uighur	2 (9%)

Abbreviation: GBMSM, gay, bisexual, and other men who have sex with men.

^a^ Other identities include pansexual and gender fluid.

In the following the five themes relating to the stages of initiation, maintenance, escalation, dependence and disengagement are presented, demonstrating the role of the five basic principles of LCT in their context.

### Initiation: First encounters with chemsex

The theme of initiation captures participants' retrospective meanings of their first chemsex experiences. These accounts were framed not as acts of deliberate pursuit, but as unplanned, socially mediated encounters within trusted networks. Rather than proactively seeking substances, most participants encountered mephedrone or α‐PHP in intimate contexts, at private group sex parties or gatherings with friends, where substance use was already normalised and embedded in collective practices of shared pleasure.

Participants described these early encounters as coincidental or opportunistic, with substances introduced casually by trusted friends or sexual partners. The principle of time and place in LCT can be clearly made out here, as the ready availability of chemsex substances in these settings and moments acts as a catalyst for first‐time use. In these contexts, the social credibility of those offering substances appeared to serve as a key mechanism of perceived risk minimisation. Narratives emphasised the emotional comfort and familiarity of the setting. As one participant explained:

‘I participated in a group sex session a few times. Then, I noticed they were using drugs. I asked what it was, they offered some […] I was curious and trusted them. I thought ‘why not’ […] I wasn't worried.’ (P6, 32 years old)

These accounts reflect the LCT principle of linked lives, whereby peer networks influence both perceived safety and initiation [40, 41]. Substances were not presented as risky, but rather as socially accepted ways of enhancing pleasure and intimacy.

‘Every one of these people I used with talked about incredible sensations. It's going to be fantastic! You can't even imagine!’ (P18, 36 years old)

Participants recalled these early experiences as being emotionally intense and sometimes transformative. For some, chemsex provided a way to relieve loneliness or unmet emotional needs. One participant memorably described this as ‘emptiness filled with love’. Substances amplified physical and emotional connection, setting a new standard for intimacy:

‘It was the kind of sex where I thought, ‘Damn, finally the sex I wanted.’ I felt confident, sexy and desirable […] It wasn't just for the sake of sex. It was an act of love.’ (P1, 28 years old)

The LCT principle of agency becomes visible in these narratives, as participants chose to use not only for an ‘improved’ sexual experience, but also to enhance their emotional experience and quality of feelings.

Initial use was often described as exploratory and limited by self‐imposed rules. Many participants framed their early use as manageable and unproblematic, emphasising their ability to control frequency, dosage and context, again highlighting agency:

‘First of all, you probably need to control yourself, in the sense that you shouldn't take too much at once, because then your head might start to hurt.’ (P12, 23 years old)

However, this sense of control was largely rooted in the absence of immediate negative consequences and the strong normative support within the social environment. Many participants later reflected that these seemingly harmless beginnings laid the foundation for more established patterns of engagement. Emotional and neurochemical associations formed during early encounters began to recalibrate expectations about sex, intimacy and emotional closeness:

‘When you're on drugs, there's more caresses, feelings, emotions […]. But when I have sex without drugs, it's colder, dry, unemotional. It's like your partner is just doing some duty and that's it.’ (P4, 24 years old)

In the context of the principle of linked lives this quote takes on another layer of meaning, as the substance use is not only affected by social networks, but also has an effect on interdependently lived lives and relationships.

These early experiences functioned as structuring events, the combination of affective intensity, peer approval and perceived safety normalised chemsex and set in motion patterns that deepened through repetition and shifting personal boundaries.

### Maintenance phase: Establishing routines and self‐imposed limits

After an initial period of experimentation, participants entered a maintenance phase characterised by self‐imposed limits and routine planning. Chemsex was viewed as a ‘reward’ or ‘holiday’, typically arranged around work or family commitments:

‘When you're using [mephedrone], you want to have fun. It's like a holiday. You plan ahead. When my mum was going to another city, I knew how long she'd be away, took time off work and planned to use.’ (P21, 26 years old)

Participants integrated use into routines aligned with work and personal responsibilities, often limiting it to weekends. These temporal boundaries served a dual function—enabling participants to meet commitments while psychologically reinforcing the belief that chemsex remained under their control.

However, several participants described a declining interest in sober sex, suggesting that chemically enhanced intimacy was becoming their new standard:

‘It [chemsex practice] made me uncomfortable to have sex when sober, both in relationships and with random guys. I felt self‐conscious when they touched me sober.’ (P11, 28 years old)

These accounts reflect early shifts in desire in which expectations of pleasure were increasingly shaped by substance's stimulation. Although rarely framed as problematic, these recalibrations suggested a deepening entanglement between substance use, sex and emotional regulation. This also reflects how desirability norms in contemporary gay culture, amplified through digital platforms, create environments where substance use becomes a strategy to mitigate feelings of inadequacy [42, 43, 44].

Emotionally significant life events could further destabilise these seemingly controlled patterns. For example, one participant described increased use following the death of his mother:

‘After my mother died, I started feeling terrible […] I distracted myself by taking mephedrone. I started using more […] 4 times a month, one gram. Four grams a month.’ (P8, 26 years old)

This illustrates the principle of timing, where the impact of bereavement during early adulthood, a critical period for emotional development, had a significant effect on substance use. Additionally, this account highlights the principle of linked lives, as the loss of a close family relationship disrupted emotional stability and coping mechanisms.

Within the life course framework, the maintenance phase is not a stable midpoint, but a transitional space where dependence begins to grow through subtle concessions as protective structures erode.

### Escalation phase: Increased frequency and quantity

The transition from controlled use to escalation was gradual, marked by the breakdown of boundaries that once offered structure. Rules around timing, dosage and context faded, often without conscious awareness. Weekend use spilled into weekdays, and brief sessions became multi‐day binges. As tolerance grew, participants increased frequency and quantity, shifting from deliberate to habitual and less controlled use aligns with the principle of timing in the life course framework, where the cumulative impact of behaviours begins to shape trajectories in more consequential ways.

‘In general, it all starts on the weekend, you go to a bar, meet someone or go to a bar with this person, and you take, one gram for the two of you. And then, you just want more, and more, and more of the drug. I mean, at first you are stimulated, the drug has an effect on you, and then you just don't want to sleep or eat, but you keep on using.’ (P15, 20 years old)

Participants attributed escalation to embodied effects, tolerance and craving, which interacted with social dynamics such as peer invitations and group norms, creating a reinforcing cycle that was pharmacological, emotional and social simultaneously.

‘I didn't plan it, but I went a whole week.’ (P20, 28 years old)

However, escalation was not only chemical; social processes were equally influential. Participants described increasing embeddedness in chemsex networks where frequent invitations, and a sense of belonging made withdrawal difficult. Although these communities were experienced as emotionally validating and protective, they also functioned as echo chambers where risk was minimised and frequent or high‐risk use was normalised:

‘Looking back, it was the only social group where I wouldn't face violence, where we used drugs and understood each other. We forgave the craziness related to drug use because we understood what everyone was going through.’ (P6, 32 years old)

This reflects the principle of linked lives, as participants' trajectories were shaped by interdependence with peers whose shared experiences reinforced ongoing substance use.

As substance use intensified, participants reported both physical and psychological symptoms, such as sleep disruption, poor nutrition, cognitive fatigue and deepening distress during comedowns. Feelings of emptiness, anxiety and self‐loathing were common. Instead of prompting cessation, these states often triggered further use to avoid emotional discomfort. The principle of life‐span development is evident here, as coping and pleasure‐seeking strategies evolved over time, increasingly centring on substance use. This cycle, using to escape the consequences of use, deepened dependence:

‘It often turns into some kind of shit, like I've fucked someone […] you come down, he comes down, he says, ‘go make another line’ and more and more […]’ (P2, 25 years old)

Growing social withdrawal from non‐using friends and family deepened reliance on chemsex networks—spaces that, in Kazakhstan's context of criminalisation and absent affirming services, remained the primary source of belonging and emotional safety.

Despite clear signs of harm, many participants maintained the belief that their use was still manageable or that the consequences were unrelated to chemsex. One participant reflected on how emotional apathy after a binge led him to quit his job, without explicitly attributing this to substance use:

‘After this binge in January, because of the apathy and withdrawal, I quit my job because, well, I didn't really like the job. […] I just said I didn't want to work and quit. It was a rash decision, wrong, because I got even more depressed because I couldn't find a job for a long time after that.’ (P21, 26 years old)

This represents a turning point—a decision shaped by emotional and physiological distress that nonetheless redirected his life course, illustrating how agency persists even under severe constraint.

Escalation rarely occurred through dramatic ruptures, but unfolded through the gradual accumulation of concessions, shifting norms and unresolved emotional needs, while participants continued to describe their use as manageable.

### Dependency phase: Loss of control and negative consequences

For some, chemsex use progressed into a phase marked by diminished agency, emotional entrapment and mounting harms across life domains. Substance use shifted from enhancing pleasure to coping with withdrawal, comedowns and emotional distress. Despite recognising the harms, participants described a growing inability to stop or reduce use, signalling a critical shift toward dependency.

‘I wanted more and more. I thought I needed more to stop the shaking. I ended up snorting 4 grams. I was all blue with terrible bags under my eyes. I don't remember how I got home.’ (P13, 21 years old)

In the life course framework, this phase marks a key turning point, when intentional use gives way to compulsion and autonomy erodes. Such transitions often unfold gradually, but have lasting consequences. Use was sustained through multi‐day binges, with little concern for sleep, nutrition or health. Sessions ended only through exhaustion, financial strain or obligations. To sustain access, participants often compromised personal values, neglected work and damaged relationships:

‘Well, lately people have even been writing me in the middle of the week, and I might just fly off and go meet them. Despite work and everything else and then just sit all day like a fucking zombie after it all.’ (P3, 34 years old)

Participants commonly reported physical health deterioration, experiencing weight loss, fatigue, sleep issues and weakened immunity. These were often accompanied by psychological distress such as anxiety, depression, paranoia and sexual dysfunction. Emotional numbness, unstable moods and intrusive thoughts also became commonplace. For some, chemsex shifted from being a source of pleasure to becoming a means of avoiding emotions or self‐medicating. One participant reflected:

‘[…] you become apathetic, start having health problems, problems with your bones, because this substance flushes calcium and magnesium from the body, and you become nervous, you constantly […] you're in search of new doses of this drug.’ (P16, 23 years old)

Social isolation deepened. Many participants withdrew from non‐using friends and family to avoid judgement, conflict or disclosure. Some lost long‐standing relationships or support systems.

‘Someone said if I continue, “I'm going to stop communicating with you” […] It's insulting when someone thinks like that. They can't help me because they see me as a hopeless addict. (P12, 23 years old)

Work performance declined, and for some, continued employment became unsustainable because of absenteeism or emotional instability. These developments again reflect the principle of time and place, especially in socio‐political contexts like Kazakhstan, where stigma is amplified and open dialogue discouraged.

As ties to non‐using networks weakened, participants relied increasingly on chemsex circles, leading to greater secrecy and reinforcing substance use. This reflects the principle of linked lives, which is the breakdown of supportive ties and growing dependence on substance‐using peers shaped both behaviour and how it was perceived as normal. Participants' choices were embedded in social worlds that constrained and enabled their trajectories simultaneously.

Although some participants identified protective factors, such as meaningful work, supportive relationships or early recognition of emerging risks, that mitigated or delayed progression, others described increased vulnerability because of pre‐existing mental health conditions, polydrug and alcohol use, financial instability or lack of non‐sexual social ties.

‘I want to tell someone that […] I'm fucked up, what am I doing? I go to the river […] I sit and cry and scream. I calm down, wipe away tears, then return as if nothing happened. Nobody knows. I go there several times a week because I have no one to talk to.’ (P20, 28 years old)

These varied outcomes underscore linked lives and the presence or absence of supportive relationships shaped participants' resilience or vulnerability. Social environments either buffered risk or deepened harm. For several, this phase ended in crisis, driven not only by internal exhaustion, but by external shocks like a friend's overdose, arrest or hospitalisation. These events marked breaking points in trajectories that had become increasingly unstable and emotionally unsustainable.

In the life course framework, such crises are key turning points, which are emotionally or structurally disruptive events that interrupt established patterns and prompt re‐evaluation [27, 36, 45]. These ruptures break through denial and trigger more serious reflection on substance use. They often arise when coping mechanisms are exhausted and previously normalised behaviours can no longer be sustained:

‘After using, since I think about my mother [who died recently], I dream about her, it gets worse. I'm completely destroyed. So, I want to quit this crap.’ (P8, 26 years old)

In some cases, the trigger was not a single crisis, but a chronic experience of loneliness and emotional disconnection that participants described as deeply painful.

‘Speed [α‐PHP] is basically every day. But I have to stop, it's not good for me. It hurts my social life. I withdraw from everyone. People don't understand when I tell them, so I end up feeling lonely […]’ (P10, 34 years old)

This illustrates the principle of linked lives, demonstrating how isolation from supportive relationships can exacerbate problematic behaviour. Without meaningful connections or empathetic responses, the social support that fosters resilience is lost. Over time, this erosion of the social network fuels continued substance use and deepens emotional abandonment, reinforcing a trajectory of dependence and disconnection.

### Disengagement phase: Recovery attempts and imagined futures

Although the dataset did not include sustained disengagement exceeding 12 months as inclusion criteria required participants to have engaged in chemsex within the past year, several participants described active attempts to reduce or cease use, revealing recursive rather than linear trajectories. Participants cycled between periods of use, temporary abstinence and renewed engagement, with each cycle shaped by the availability or absence of structural support. At the time of interview, one participant had achieved nearly 4 months of continuous abstinence, having accessed private treatment that included medical detoxification, psychotherapy using hypnotherapy techniques and comprehensive nutritional rehabilitation:

‘I've been clean for 118 days. During hypnotherapy I suddenly found myself laughing—the first time I'd heard my own laughter this year.’ (P6, 32 years old)

This account reveals how access to affirming clinical care that addressed dependence symptoms and underlying psychological distress made recovery possible. However, it also exposes profound inequalities, for example, this participant received private treatment unavailable to most others in the sample and recovered within supportive social networks that many participants lacked entirely. The principle of linked lives is evident here, as recovery trajectories depended not only on individual motivation, but also on the presence of relationships and resources that could prevent relapse. Another participant achieved 6 months of abstinence following a life‐threatening medical crisis that required 3 months of hospitalisation in an induced coma:

‘I spent 3 months in hospital from August, so I couldn't use drugs. After that, I remained abstinent until February and didn't experience cravings or withdrawal.’ (P10, 34 years old)

This trajectory shows how forced abstinence through medical intervention can disrupt established patterns of use. Interestingly, this participant initially reported no withdrawal symptoms, but returned to daily use approximately 6 months later suggesting that abstinence achieved through external circumstances rather than active recovery may be difficult to sustain without ongoing support. Not all participants sought total abstinence. One participant, abstinent for 4 months, explicitly rejected permanent disengagement, instead envisioning a future relationship with drugs characterised by control and distance:

‘It feels like I'm no longer a student but a teacher. I can control some of my demons now, maybe even keep them at a distance—continue, but from afar and on better terms.’ (P5, 26 years old)

This reflects a harm reduction approach, where reducing risks and maintaining control are the primary objectives rather than achieving abstinence. Similarly, a participant who had been abstinent for 6 months did not view potential relapse as failure, but as an expected part of ongoing management:

‘I look at it medically; remission is always possible. I mean, remission is also normal, I mean, after remission, you go back to not using […] Remission usually doesn't last long.’ (P8, 20 years old)

His medicalised approach normalised cyclical patterns of use and abstinence, reflecting the understanding that recovery processes are rarely linear. For others, however, the idea of quitting remained entirely dependent on experiencing acute risks. One participant expressed belief in his capacity to quit, while also acknowledging his current unwillingness:

‘I think I could still quit mephedrone if I wanted to. At one point, I wanted to quit because it's only temporary. Then I thought it was still too early.’ (P3, 34 years old)

This conditional framing, maintaining the belief that cessation remains possible while continuing to use, enabled the continuation without confronting dependence. The absence of sustained disengagement in this sample reflects not only barriers to recovery, but also diverse relationships with substance use, where abstinence is neither universally desired nor prioritised.

Within the LCT framework, these narratives demonstrate that trajectories are dynamic processes shaped by turning points, the quality of available support and individual goals, rather than predetermined progressions. The principle of time and place is central, and recovery requires structural transformation, including decriminalisation and the expansion of LGBTQ‐affirming addiction care that respects diverse approaches to managing substance use, as well as individual motivation.

## DISCUSSION

This study applied LCT to examine chemsex trajectories among GBMSM in Almaty. Our most significant finding is that, in criminalised contexts, structural violence—the intersection of drug criminalisation and homophobia rather than individual pathology, determines trajectories. The absence of sustained disengagement over longer time periods in our sample indicates that recovery is structurally challenging rather than reflecting individual failure, challenging individualistic addiction frameworks.

### Structural violence emerges as the primary driver of trajectories

Our LCT analysis conceptualises three mechanisms through which criminalising policies and pervasive homophobia generate and sustain problematic substance use patterns. Initiation consistently occurred through trusted social networks rather than individual risk‐seeking behaviour documented in Western contexts. Escalation was driven by the accumulation of unaddressed trauma and the erosion of support systems for those not using substances. Finally, structural barriers prevented help‐seeking without risking imprisonment and lifelong exclusion. These findings are consistent with scholarship on ‘structural violence’ [46, 47, 48, 49] and build on it by demonstrating how drug criminalisation intersects with sexual stigma to create conditions in which substance use becomes a survival strategy. Our findings support calls for ‘structural interventions’ [47]: decriminalisation, legal protections and affirming care are foundational pre‐requisites for recovery, not supplementary measures. The participant with the longest continuous abstinence accessed private treatment unavailable to others, exemplifying how structural inequalities determine recovery outcomes.

### Divergence from Western chemsex research: Double marginalisation and network functions

Our findings indicate fundamental differences in chemsex trajectories between Kazakhstan and high‐income Western settings, where LGBTQ+ individuals typically have access to community infrastructure, legal protections, harm reduction services and social acceptance [1, 3, 50, 51]. In those contexts, chemsex may serve as enhancement or escape and support is theoretically accessible when use becomes problematic. However, the intersection of drug criminalisation with homophobia, what scholars term ‘double marginalisation’ [48, 52], creates qualitatively different conditions that reshape the meaning and function of chemsex networks. These networks functioned as survival mechanisms rather than merely social spaces, often being the only environments in which participants could avoid violence, experience intimacy and find mutual understanding. This explains why peer networks remained emotionally central even during escalation and dependence, diverging from Western research that documents social withdrawal as a marker of problematic use.

The dual nature of these networks, simultaneously protective and risk‐amplifying, poses challenges for intervention design. Participants rely on chemsex networks precisely because mainstream society offers no affirming spaces. Yet these same networks may normalise escalating use and reinforce isolation from potential sources of help. Although Western interventions often aim to reduce network embeddedness and increase connections to non‐using support networks, this approach is inapplicable where such networks do not exist. Western frameworks that distinguish between ‘recreational’ and ‘problematic’ use or frame recovery exclusively as abstinence, require substantial adaptation for contexts in which substance use is inextricably linked to meeting basic needs such as safety and belonging. These differences suggest that chemsex trajectories are deeply context‐dependent and shaped by the specific configuration of structural forces in each setting.

### Challenging linear progression and abstinence‐centred frameworks

Rather than following a linear progression, participants' trajectories were characterised by recursive cycling between phases, temporary cessation followed by renewed use and diverse patterns that did not align with the narrative of ‘progression to dependence’. Some maintained controlled use for extended periods, whereas others experienced rapid escalation following specific life events. Others cycled between dependence and temporary abstinence without achieving sustained disengagement. This variability is consistent with longitudinal substance use research [24, 25] and shows that progression is neither inevitable nor uniform. LCT offered a more comprehensive explanation than traditional stage models by showing how critical junctures, bereavement, relationship dissolution, health crises, could trigger either escalation or disengagement attempts depending on the availability of support and how participants exercised fragile, but persistent self‐regulation even within severe constraints. These findings support critiques of addiction models that overlook the social and emotional dimensions of substance use [53, 54, 55] and position behavioural change as an individual rather than a structural problem.

Several participants explicitly rejected total abstinence as their recovery goal, instead articulating harm reduction approaches that prioritised control, distance or managed use. One participant's metaphor of transitioning from ‘student’ to ‘teacher’ in relation to substances reflected agency and self‐knowledge rather than denial. Another participant's medicalised framing of ‘remission’ normalised cyclical patterns and rejected binary definitions of success versus failure. These recovery goals were not merely expressions of harm minimisation, but rather realistic assessments of what could be achieved within their structural realities. This challenges the dominance of abstinence‐only frameworks in treatment systems, particularly in post‐Soviet contexts where coercive, abstinence‐based models prevail [56, 57, 58]. Imposing abstinence‐only definitions in contexts where sustained abstinence is not structurally supported may alienate individuals seeking help and reinforce the stigma that drives substance use underground. Therefore, interventions must respect diverse recovery goals and recognise that harm reduction may be the only viable approach in contexts lacking affirming care and structural support.

### Theoretical implications: Reconceptualising LCT

Although LCT provided valuable analytical tools, this study demonstrates that theoretical frameworks developed in Western, heteronormative contexts require substantial reconceptualisation when applied to marginalised populations under extreme structural constraints. Our analysis reveals three interconnected limitations.

The principles of agency and linked lives both operated in ways that contradicted typical assumptions of LCT. Participants made deliberate choices, such as setting rules, seeking moderation and attempting recovery. However, these efforts were so severely constrained that traditional understandings of ‘choice’ or ‘autonomy’ proved inadequate. What we observed was ‘bounded agency’, and under extreme structural violence, agency may be reduced to navigating degrees of harm rather than pursuing positive outcomes. Similarly, LCT generally conceptualises social relationships as sources of support that shape trajectories toward normative outcomes. However, when normative support systems are inaccessible because of structural exclusion, relationships simultaneously function as survival mechanisms and risk amplification systems. This dual nature presents challenges for frameworks that assume relationships operate within contexts where normative support is available.

The interaction of time and place with other principles created conditions that LCT does not adequately theorise. Our findings demonstrate how structural violence compresses temporal dynamics and forecloses developmental possibilities, for instance, participants experienced rapid transitions between phases, urgency in decision‐making that prevented long‐term planning and an absence of institutional support for behavioural change. The absence of sustained disengagement in our sample is not merely an observation about barriers to recovery, but evidence that extreme structural violence creates fundamentally different developmental conditions to those typically described by LCT. These findings suggest that LCT requires theoretical reconceptualisation rather than flexible application. Future applications must explicitly theorise how structural violence transforms core principles, rather than assuming that acknowledging constraints is sufficient. Life course development in contexts of extreme marginalisation may follow substantially different patterns to those documented in populations with access to normative pathways and institutional support.

### Limitations

This study has several important limitations. The cross‐sectional design relied on retrospective accounts subject to recall bias, and as all participants had engaged in chemsex within the last 12 months, long‐term trajectories and sustained disengagement could not be examined. Selection bias likely influenced the sample. Recruitment through community organisations may have favoured socially connected, younger, urban individuals already using services, while under‐representing isolated individuals, those from other regions, older GBMSM and those fully disengaged. Participants experiencing problems may have been more motivated to participate, potentially overestimating harm, while social desirability bias may have influenced narratives in either direction. The lead author's shared identity facilitated rapport, but his evolving understanding of harm, following a friend's death during analysis, may have unconsciously shaped interpretation. The study was limited to Russian‐speaking participants in Almaty. Finally, although we argue for reconceptualising LCT, our analysis remained constrained by a framework developed in Western contexts and may not have fully captured patterns specific to Central Asian queer life‐worlds.

### Policy and practice implications

These findings have important implications for policy and practice. Structural reforms that decriminalise drug use and protect LGBTQ+ rights are pre‐requisites for meaningful public health responses. As long as seeking help carries the risk of imprisonment and lifelong exclusion, individual‐level interventions will remain severely limited. Harm reduction services must expand beyond HIV prevention to include trauma‐informed mental health support, recognising that participants primarily used substances for emotional regulation and survival rather than sexual enhancement. LGBTQ‐affirming mental healthcare must form a central part of chemsex interventions. Peer‐led interventions delivered via digital platforms currently used to organise chemsex could leverage existing trust networks and reach individuals who avoid formal services, although peer interventions alone cannot compensate for structural violence and must be accompanied by policy reforms addressing root causes.

### Future research

Future research should explore later stages of the chemsex trajectory, particularly recovery, through longitudinal designs that can capture sustained disengagement patterns not present in this sample. Such research could investigate factors that enable some individuals to maintain or regain control, such as stable employment, family support, or affirming partnerships. Comparative research across EECA contexts and between EECA and Western settings would test whether theoretical frameworks are portable and illuminate how configurations of criminalisation, healthcare access and LGBTQ+ rights shape trajectories. Research examining the impact of structural reforms could inform policy advocacy. Finally, participatory research involving people with lived experience at every stage of the research process would prioritise community knowledge and ensure that the findings reflect the priorities and realities of those most affected by chemsex‐related harms.

## AUTHOR CONTRIBUTIONS

**Nikolay Lunchenkov:** Conceptualization (lead); data curation (lead); formal analysis (equal); funding acquisition (supporting); investigation (lead); methodology (equal); project administration (supporting); resources (supporting); software (equal); writing—original draft (lead). **Nadezhda Cherchenko:** Conceptualization (supporting); investigation (supporting); methodology (supporting); resources (equal); validation (equal); writing—original draft (supporting); writing—review and editing (supporting). **Elena German:** Conceptualization (supporting); funding acquisition (lead); project administration (equal); resources (lead); writing—original draft (supporting); writing—review and editing (supporting). **Susanna Rinne‐Wolf:** Conceptualization (equal); data curation (equal); formal analysis (supporting); supervision (lead); writing—original draft (equal); writing—review and editing (equal).

## DECLARATION OF INTERESTS

None.

## Supporting information


**Data S1.** Supporting Information.

**Data S2.** Supporting Information.

**Data S3.** Supporting Information.

**Data S4.** Supporting Information.

## Data Availability

Data available on request due to privacy/ethical restrictions.
