# Supplementary material for: ‘Emptiness filled with love’: A reflexive thematic analysis of chemsex trajectories among gay, bisexual and other men who have sex with men in Almaty, Kazakhstan, using a life course framework
Source: Addiction. 2026 Apr 24;121(9):2532–44. doi: 10.1111/add.70454 (PMC13486950; doi:10.1111/add.70454)
Supplement: Supplementary file 1 [file ADD-121-2532-s001.pdf]

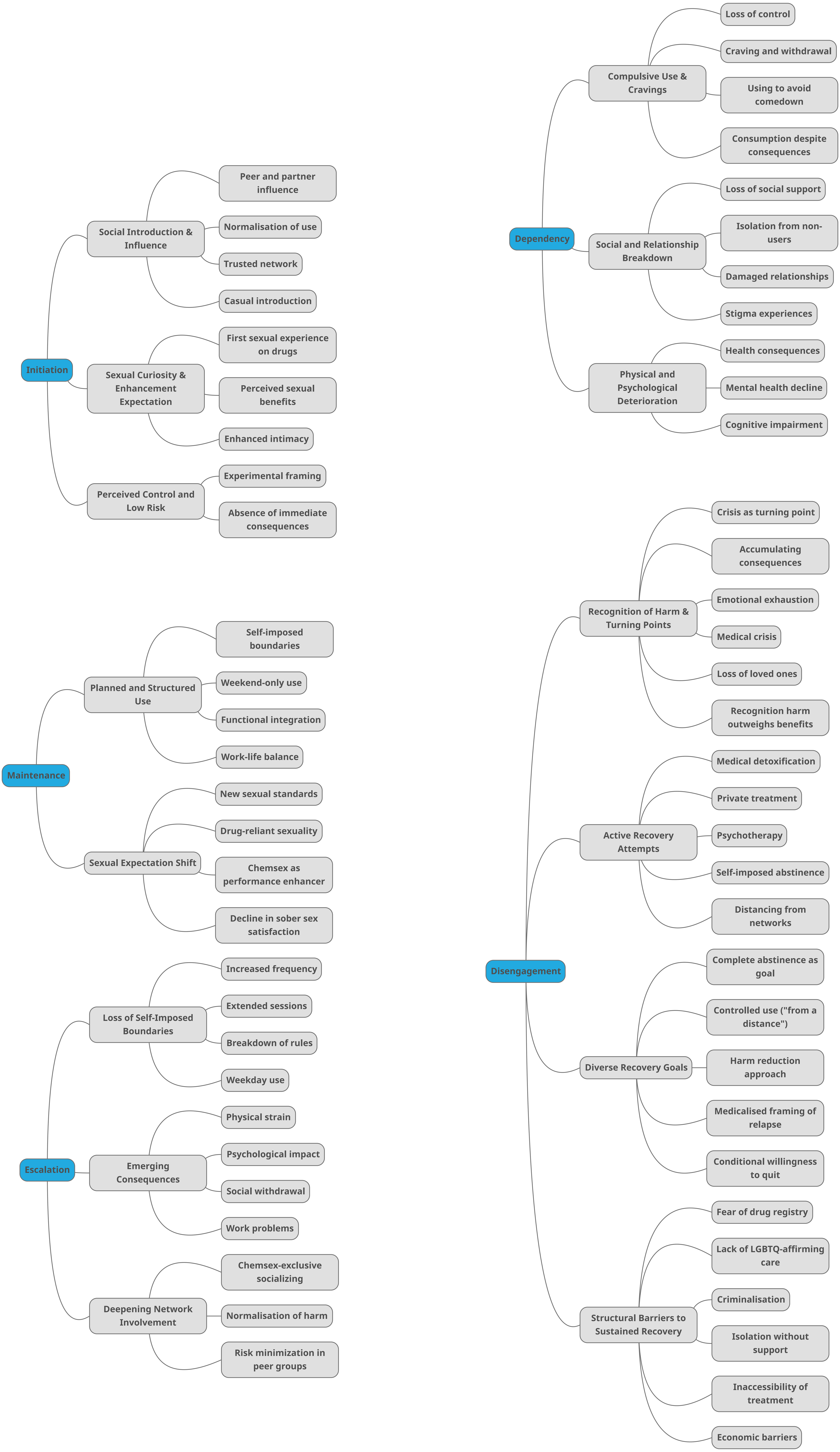
Initiation
Social Introduction & Influence
Peer and partner influence
Normalisation of use
Trusted network
Casual introduction
Sexual Curiosity & Enhancement Expectation
First sexual experience on drugs
Perceived sexual benefits
Enhanced intimacy
Perceived Control and Low Risk
Experimental framing
Absence of immediate consequences
Maintenance
Planned and Structured Use
Self-imposed boundaries
Weekend-only use
Functional integration
Work-life balance
Sexual Expectation Shift
New sexual standards
Drug-reliant sexuality
Chemsex as performance enhancer
Decline in sober sex satisfaction
Escalation
Loss of Self-Imposed Boundaries
Increased frequency
Extended sessions
Breakdown of rules
Weekday use
Emerging Consequences
Physical strain
Psychological impact
Social withdrawal
Work problems
Deepening Network Involvement
Chemsex-exclusive socializing
Normalisation of harm
Risk minimization in peer groups
Dependency
Compulsive Use & Cravings
Loss of control
Craving and withdrawal
Using to avoid comedown
Consumption despite consequences
Social and Relationship Breakdown
Loss of social support
Isolation from non-users
Damaged relationships
Stigma experiences
Physical and Psychological Deterioration
Health consequences
Mental health decline
Cognitive impairment
Disengagement
Recognition of Harm & Turning Points
Crisis as turning point
Accumulating consequences
Emotional exhaustion
Medical crisis
Loss of loved ones
Recognition harm outweighs benefits
Active Recovery Attempts
Medical detoxification
Private treatment
Psychotherapy
Self-imposed abstinence
Distancing from networks
Diverse Recovery Goals
Complete abstinence as goal
Controlled use ("from a distance")
Harm reduction approach
Medicalised framing of relapse
Conditional willingness to quit
Structural Barriers to Sustained Recovery
Fear of drug registry
Lack of LGBTQ-affirming care
Criminalisation
Isolation without support
Inaccessibility of treatment
Economic barriers
