# Supplementary material for: ‘Emptiness filled with love’: A reflexive thematic analysis of chemsex trajectories among gay, bisexual and other men who have sex with men in Almaty, Kazakhstan, using a life course framework
Source: Addiction. 2026 Apr 24;121(9):2532–44. doi: 10.1111/add.70454 (PMC13486950; doi:10.1111/add.70454)
Supplement: Supplementary file 2 [file ADD-121-2532-s002.pdf]

Supplementary file 2 – Interview guide

Opening questions

1) Tell me a bit about yourself.

- What hobbies/interests do you have?

- What do you do on a weekend?

- Do you like to socialize or spend time alone?

- Do you like going to Parties?

- Do you use any dating apps to get to know people, e.g. Grinder?

- How would you describe your sexuality to someone you trust?

Sexual behavior

2) Could you share with me some more about your first sexual experiences?

- How old were you?

- Who was the partner? Someone you knew or someone you didn't know?

Age? Gender?

3) Do you experience any negative emotions after you have sex with a man? If yes, what are they?

Prompts: guilt, shame, etc.

- How do you deal with these emotions?

- Prompts: talk to someone, therapy, distraction, partying/drugs, etc.

Chemsex

4) Could you describe your last sexual experience with a man?

- Types of sexual activities

- How many people involved

- If drugs are mentioned:

- Which drugs did you use?

- How do you agree on which drugs to use?

- Where did it happen? Tell me more about physical location.

If drugs are not mentioned:

- Ask about sexual experience with drug use and then follow to Chemsex questions.

5) How does engaging in drug use and sex/chemsex make you feel?

- Motivation

- Physical feelings during and after.

- Emotional feelings during and after.

6) How often do you use dating apps when looking for chemsex partners/parties?

If they use dating apps:

- Which apps

- What do apps help you with that would otherwise be difficult

- Do you feel using these apps is safe?

- What role do these apps play in the rest of your sex life? Can you meet someone outside of the apps?

Risk perception and services experience

7) Can you tell me more about your recent experience with sexual health/HIV/drugs services

- Which services do you usually use and what for?

- How were you treated by medical professionals?

- Did you receive any harm reduction materials (needles)? If yes: What harm reduction materials did you receive? Condoms, needles etc

- How did this experience differ from other times you went to a clinic?

8) Did you talk with your doctor about Chemsex? If yes: what did you discuss?

Have you ever experienced any problems or had bad experiences using drugs?

- If no: Have you experienced gay friends have problems with drugs?
- If yes:
- Please describe the experiences.
- How did you cope?

Closing questions

9) What motivated you to come and be interviewed today?

10) Do you have any questions or is there any aspect that we did not cover in our interview that you would like to mention/discuss further?
