# Supplementary material for: ‘Emptiness filled with love’: A reflexive thematic analysis of chemsex trajectories among gay, bisexual and other men who have sex with men in Almaty, Kazakhstan, using a life course framework
Source: Addiction. 2026 Apr 24;121(9):2532–44. doi: 10.1111/add.70454 (PMC13486950; doi:10.1111/add.70454)
Supplement: Supplementary file 3 [file ADD-121-2532-s003.pdf]

Supplementary file 4 – Deductive Framework and Inductive Coding Structure

Trajectory Stage (Deductive)	Inductive Subthemes	Example Inductive Codes	LCT Principles Applied as Analytical Lenses
**Initiation**	Social Introduction & Influence	Peer and partner influence; Normalization of use; Trusted network; Casual introduction	Linked Lives, Time and Place
	Sexual Curiosity & Enhancement	First sexual experience on drugs; Expectation of pleasure; Perceived sexual benefits; Enhanced intimacy	Agency, Lifespan Development
	Perceived Control and Low Risk	Experimental framing; Self-imposed limits; Absence of immediate consequences	Agency
**Maintenance**	Planned and Structured Use	Self-imposed boundaries; Weekend-only use; Functional integration; Work-life balance	Agency, Time and Place
	Sexual Expectation Shift	Drug-reliant sexuality; Chemsex as performance enhancer; Decline in sober sex satisfaction; New sexual standards	Lifespan Development, Linked Lives
**Escalation**	Loss of Self-Imposed Boundaries	Increased frequency; Extended sessions; Breakdown of rules; Weekday use	Timing, Agency
	Emerging Consequences	Physical strain; Psychological impact; Social withdrawal; Work problems	Linked Lives, Time and Place
	Deepening Network Involvement	Chemsex-exclusive socializing; Normalization of harm; Risk minimization in peer groups	Linked Lives
**Dependency**	Compulsive Use & Cravings	Loss of control; Craving and withdrawal; Using to avoid comedown; Consumption despite consequences	Agency, Timing
	Social and Relationship Breakdown	Loss of social support; Isolation from non-users; Damaged relationships; Stigma experiences	Linked Lives, Time and Place

Trajectory Stage (Deductive)	Inductive Subthemes	Example Inductive Codes	LCT Principles Applied as Analytical Lenses
	Physical and Psychological Deterioration	Health consequences; Mental health decline; Cognitive impairment	Lifespan Development
	Recognition of Harm & Turning Points	Crisis as turning point; Accumulating consequences; Emotional exhaustion; Medical crisis; Loss of loved ones; Recognition harm outweighs benefits	Timing, Agency
	Active Recovery Attempts	Medical detoxification; Private treatment; Psychotherapy; Self-imposed abstinence; Distancing from networks	Linked Lives, Time and Place
**Disengagement**	Diverse Recovery Goals	Complete abstinence as goal; Controlled use ("from a distance"); Harm reduction approach; Medicalized framing of relapse; Conditional willingness to quit	Agency, Lifespan Development
	Structural Barriers to Sustained Recovery	Fear of drug registry; Lack of LGBTQ-affirming care; Criminalization; Isolation without support; Inaccessibility of treatment; Economic barriers	Time and Place, Linked Lives
